# The Physician’s All-Requisite Time and Labor-Saving Account Book

**Published:** 1891-01

**Authors:** 


THE PHYSICIAN'S ALL-REQUISITE TIME AND LABOR-SAVING ACCOUNT-BOOK: Being a ledger and account-book for Physicians' use, meeting all the requirements of the law and courts.

Probably no class of people lose more money through carelessly kept accounts and over-looked or neglected bills than the physician. Often detained at the bedside of the sick until late at night, or deprived of even a modicum of rest, it is with great difficulty that he spares the time or puts himself in condition to give the same care to his own financial interests that a merchant, a lawyer, or even a farmer devotes. It is plainly apparent that a system of book-keeping and accounts that, without sacrificing accuracy, but, on the other hand, insuring it, at the same time relieving the keeping of a physician's books of half their complexity and two-thirds the labor, is a convenience which will be eagerly welcomed by thousands of overworked physicians. Such a system has at last been devised, and it is offered to the profession in the form of *The Physician's All-Requisite Time and Labor-Saving Account-Book.* A few of the superior advantages of *The Physician's All-Requisite Time and Labor-Saving Account-Book*, are as follow: 1. Will meet all the requirements of the law and courts. 2. Self-explanatory; no cipher code. 3. Its completeness without sacrificing anything. 4. No posting; one entry only. 5. Universal; can be commenced at any time of year, and can be continued indefinitely until every account is filled.

No. 1. 300 pages for 900 accounts per year, $5.00. F. A. Davis, 1231 Filbert Street, Philadelphia, Pa.

